# Life span, growth, senescence and island syndrome: Accounting for imperfect detection and continuous growth

**DOI:** 10.1111/1365-2656.13842

**Published:** 2022-11-17

**Authors:** Andreu Rotger, Simone Tenan, José‐Manuel Igual, Simon Bonner, Giacomo Tavecchia

**Affiliations:** ^1^ Animal Demography and Ecology Unit, GEDA – IMEDEA (CSIC/UIB) Esporles Spain; ^2^ MUSE – Science Museum, Corso del Lavoro e della Scienza Trento Italy; ^3^ National Research Council, Institute of Marine Sciences (CNR‐ISMAR) Venezia Italy; ^4^ University of Western Ontario London Ontario Canada

**Keywords:** Bayesian framework, body size, capture–recapture, indeterminate grower, life‐history traits, longitudinal data, mortality

## Abstract

1. Small vertebrates on islands are expected to attain a larger body size, and a greater survival than their mainland counterparts. Comparative studies have questioned whether lizards exhibit this set of adaptations, referred to as the ‘island syndrome’.
2. We collected data on 730 individuals the endemic Lilford's lizard *Podarcis lilfordi* throughout a 10‐year period on a small island of the Balearic archipelago (Spain)*.* We coupled a growth function with a capture–mark–recapture model to simultaneously estimate size‐ and sex‐dependent growth rate and survival. To put our results into a wider context, we conducted a systematic review of growth, life span and age at maturity in different *Podarcis* species comparing insular and mainland populations.
3. We found a low average growth coefficient (0.56 and 0.41 year^−1^ for males and females to reach an asymptotic size of 72.3 and 65.6 mm respectively), a high annual survival probability of 0.81 and 0.79 in males and females, and a large variability between individuals in growth parameters.
4. Survival probability decreased with body size in both sexes, indicating a senescence pattern typical of long‐lived species or in populations with a low extrinsic mortality. Assuming a constant survival after sexual maturity, at about 2 years old, the average life span was 6.18 years in males and 8.99 in females. The oldest animal was a male last captured at an estimated age of ≥13 years and still alive at the end of the study.
5. Our results agree with the predictions of the ‘island syndrome’ for survival, life span and growth parameters. A comparative analysis of these values across 29 populations of 16 different species of *Podarcis* indicated that insular lizards grow slower and live longer than their mainland counterparts. However, our data differed from other island populations of the same species, suggesting that island‐specific characteristics play an additional role to isolation.
6. Within this study we developed an analytical approach to study the body size‐dependent survival of small reptiles. We discuss its applicability to contrast hypotheses on senescence in different sexes of this species, and provide the code used to integrate the growth and capture–mark–recapture models.

## Graphical abstract

This work integrates growth and capture–mark–recapture models, and uses model selection analysis, to contrast hypotheses on senescence and size‐dependent survival in continuous growers.

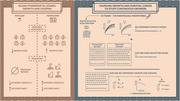

## 1 INTRODUCTION

The term ‘island syndrome’ refers to the particular set of morphological, evolutionary and behavioural changes that insular individuals undergo to adapt to their confined environment (Adler & Levins, 1994). Among the most important changes that characterize the island syndrome are a longer life span, a low fecundity and an increase in the age at first reproduction compared to mainland counterparts (Grant, 1998). Adaptations predicted by the island syndrome, have been found in insular mammals (Adler & Levins, 1994; Jordana et al., 2012), and birds (Blondel et al., 2002; Covas, 2012) in multiple traits, but recent comparative evidence has questioned whether this set of changes also occurs in small reptiles (Itescu et al., 2014; Novosolov et al., 2013; Raia et al., 2010). Comparative studies often consider the maximum value ever recorded of a given trait (e.g. body size or life span) for the species (Gibbons, 1987; Scharf et al., 2015). By definition, this measure refers to a single individual, often kept in captivity, and cannot be taken as representative of an entire population, even less of a species (Gibbons, 1987; Tidière et al., 2016). For example, Stark et al. (2018) found that the maximum longevity in reptiles is positively correlated with the number of individuals examined. Small lizards have a life expectancy that rarely exceed a few years (Andrews & Nichols, 1990; Schoener & Schoener, 1982). Mortality patterns in wild lizard populations, however, remain largely unknown (Losos, 2009; Massot et al., 2011). In predatory‐limited populations young lizards are thought to have a higher mortality than older animals (Losos, 2009; Massot et al., 2011; Vitt & Caldwell, 2013), whereas in predatory‐free populations the mortality probability is expected to increase with age (i.e. senescence). Despite these predictions, the scarce information available suggests that survival is constant throughout a lizard's life span (Andrews & Nichols, 1990; Schoener & Schoener, 1982; but see Massot et al., 2011). Whether this pattern is common to many lizard populations is poorly known (Losos, 2009) because size‐ or age‐dependent patterns in wild reptiles are difficult to describe (Massot et al., 2011). First, there is a lack of longitudinal individual‐based data on lizards (Nussey et al., 2008) and when available, senescence is often masked by the high extrinsic mortality (e.g. predation; Patnaik, 1994; Vaupel et al., 2004). In addition, the demography of indeterminate growers is likely to depend more on body size than age (Colchero & Schaible, 2014).

Demographic models built on a continuous time‐dependent variable are more complicated than those built on discrete stages as commonly used for mammals and birds (Caswell, 2000; Easterling et al., 2000). Moreover, methods to estimate survival in wild populations rarely incorporate time‐varying continuous individual covariates, such as body size, because these variables cannot be observed when an individual is not captured (Lebreton et al., 1992). To solve this problem Bonner and Schwarz (2006) proposed to model the distribution of covariates over time and between individuals according to a Gaussian random walk. When recoveries are available a second option is to condition the likelihood on the observed values (Catchpole et al., 2008). While this approach avoids having to model the covariate distribution, it requires high capture and recovery probabilities, both independent from the covariate. Coupling a capture–mark–recapture (CMR) and a body growth model has recently been proposed as a method to overcome these problems. This can be done in a Bayesian statistical framework to ‘fill‐in’ the missing values of the covariate due to recapture failures (Eaton & Link, 2011; Rose et al., 2018; Schofield et al., 2013).

Here, we used individual‐based information collected over 10 years on a small insular population of the Lilfordi's lizard, *P. lilfordi*, to estimate males and females size‐dependent annual survival, average life span and growth patterns. We infer body size growth parameters and survival patterns of insular male and female lizards in order to assess whether their values and patterns agree with the predictions of the island syndrome. We expect to find a long life span, a slow growth rate and evidence of senescence by age due to the low extrinsic mortality (Jordana et al., 2012). Predictions of sex‐dependent patterns are more difficult to formulate (Cayuela et al., 2020). Insular populations of lizards usually attain high densities and high levels of intrasexual competition, particularly in males (Raia et al., 2010). We thus predict that males grow faster than females and that senescence should be more pronounced due to the investment associated with a fast‐initial growth. However, if reproductive investment is high, female could show a more pronounced senescence pattern (Massot et al., 2011; Olsson & Shine, 2002). The integration, within a Bayesian framework, of an individual growth model with a hierarchical CMR model provides a flexible solution to the problem of missing covariates (Bonner & Schwarz, 2006; Eaton & Link, 2011; Schofield et al., 2013). Moreover, this hierarchical model offers a solid framework for the study of demographic processes in species with continuous growth, allowing for the simultaneous estimation of growth and survival, which represent fundamental parameters for life‐history studies (Caruso & Rissler, 2019; Reinke et al., 2020; Rose et al., 2018).

## 2 MATERIALS AND METHODS

### 2.1 Data collection and integrated analysis

Individual‐based data were collected on a small (0.30 ha) islet off the southern coast of Mallorca, Spain (39°16′46″N, 3°2′10″E). The islet is predator free, but the vegetation is scarce, which reduces the availability of pollinators and fruits (see Rotger et al., 2021; Ruiz de Infante et al., 2013), which are typically found in the diet of Lilford's wall lizards in other populations (Pérez‐Cembranos et al., 2016; Santamaría et al., 2020). Three‐occasion capture–recapture sessions were conducted each October from 2010 to 2019 (10 sessions) within a period of 3–6 days (see details in Ruiz de Infante et al., 2013; Tenan et al., 2013). Lizards were caught on a grid of 26 pitfall traps positioned at an average distance of 3.5 m and visited every 30 min (commonly one to three visits per session). Captured animals were measured (snout‐to‐vent length, hereafter ‘SVL’) and sexed (Rotger et al., 2016). Captured lizards were photographed prior to release and individually identified by the unique pattern of their ventral scales (Pellitteri‐Rosa et al., 2010) using the software APHIS (Moya et al., 2015). All animals were released after taking the picture in the same place where they were captured. Authorization for capturing individuals in the field was provided yearly by the Government of Balearic Islands (Ref: CEP 06/2010‐2021). Ethical approval was not required for this research.

Individual measures and capture histories were used in a single analysis, coupling a model for body growth with a model for time‐dependent survival and recapture probabilities (the Cormack‐Jolly‐Seber model; CJS, Lebreton et al., 1992). The growth model was built to impute a probability distribution for the SVL each time that a lizard, estimated to be alive, was not captured. We included apparent negative growth increments to account for measurement errors (Rotger et al., 2016). The analysis was implemented into a Bayesian framework to allow for a hierarchical modelling of body growth, survival and recapture probability simultaneously and the estimation of parameter credible interval around the average of derived parameters (e.g. life span, see below). Derived parameters are estimated with MCMC computing the value for each individual on each iteration of the chain. In addition, the Bayesian framework utilized allowed to easily include animals of unknown sex, which will be estimated by the model.

### 2.2 Body size growth

To infer the individual size from capture–recapture data of lizards of unknown ages we modelled the growth of each individual according to Schnute's growth curve (Rotger et al., 2021; Schnute, 1981), a modification of the classical von Bertalanffy's growth function (Fabens, 1965) in which parameters are less correlated (Gallucci & Quinn, 1979; Rotger et al., 2016, 2021). We used the mark–recapture, or Baker's form of the Schnute model (Baker et al., 1991). Let $L_{i,t}$ denotes the true body size of individual i in year t and $L_{i,t+D}$ represents the true body size in year t+D. For each individual, the curve is defined in terms of the individual's growth coefficient, $K_{i}$, and the size of the individual, $L_{iT_{1}}$ and $L_{iT_{2}}$, at any two time points (Baker et al., 1991). For convenience, we took the first time point to be $T_{1}=0$, so that $L_{iT_{1}}$ represents the size of individual i at birth. We used the average SVL of hatchling lizards showed in Rotger et al. (2020) and their variance as a prior information. We include an individual random effect in growth parameters to account for individual variability. The second time point $T_{2}$ is somewhat arbitrary, and we set it at age 14 years, which corresponds to the average longevity records for this species in captivity (Scharf et al., 2015). Then, the size of the individual *i* at time t+D given its size at time t is calculated as:

(1) $$L_{i,t+D}=L_{i,t}e^{-K_{i}D}+\left[L_{iT_{2}}-L_{iT_{1}}e^{-K_{i}\left(T_{2}-T_{1}\right)}\right]\frac{\left(1-e^{-K_{i}D}\right)}{\left(1-e^{-K_{i}\left(T_{2}-T_{1}\right)}\right)}.$$

We assumed that the size of an individual is measured with error such that the observed size if individual i is captured on occasion t is:

$$L_{i,t}^{\mathrm{obs}}=L_{t}+\epsilon_{\mathrm{it}}^{\left(L\right)}.$$

Errors are assumed to be independent and normally distributed with mean 0 and constant variance, $\sigma_{L}^{2}$, such that $\epsilon_{\mathrm{it}}^{\left(L\right)}∼N\left(0,\sigma_{L}^{2}\right)$.

### 2.3 Derived parameters

Several derived parameters can be computed from Schnute's model (Schnute & Fournier, 1980). The individual asymptotic size ($L_{\infty i}$) can be computed as:

(2) $$\;L_{\infty i}=\frac{L_{iT_{2}}-L_{iT_{1}}e^{-K_{i}\left(T_{2}-T_{1}\right)}}{1-e^{-K_{i}\left(T_{2}-T_{1}\right)}}.$$

To estimate age at a given SVL ($L_{i,t})$, we re‐arranged the Schnute model (equation 1) to predict age ($A_{\mathrm{Li}}$):

(3) $$A_{\mathrm{Li}}=\frac{\ln\left(\;1-\left[1-e^{-K_{i}\left(T_{2}-T_{1}\right)}\cdot \frac{L_{i,t}-L_{iT_{1}}}{L_{iT_{2}}-L_{iT_{1}}}\right]\;\right)}{-K_{i}}+T_{1}.$$

Information on the minimum size at maturity reported by Castilla and Bauwens (2000) and Galán (2003) was used to calculate the age at sexual maturity in females using Equation 3 (50 mm). For males, we used the SVL of the smallest male observed displaying secondary sexual characteristics (femoral pores; 59 mm).

### 2.4 Hierarchical models for survival

In capture–recapture models, survival and detection parameters were assumed to depend on sex and individual body size (see Bonner & Schwarz, 2006; Schofield & Barker, 2011) while time was considered to be a random component of parameter variability. The general model for survival, $\phi_{\mathrm{it}}$, was an extension of the classical CJS model with two groups, males and females. In addition, we included body size, and the interaction terms between sex and body size as explanatory covariates:

(4) $$\begin{array}{c} \text{logit}\left(\phi_{i,t}\right)=\alpha_{s}+\beta_{s1}\cdot {\mathrm{sex}}_{i}+\beta_{s2}\cdot {\mathrm{SVL}}_{i,t}+\beta_{s3}\cdot {\mathrm{SVL}}_{i,t}^{2}\\ \;+\beta_{s4}\cdot {\mathrm{sex}}_{i}\cdot {\mathrm{SVL}}_{i,t}+\beta_{s4}\cdot {\mathrm{sex}}_{i}\cdot {\mathrm{SVL}}_{i,t}^{2}+\varepsilon_{t}^{\left(\phi \right)}, \end{array}$$

where $\mathrm{SV}L_{\mathrm{it}}$ represents the standardized SVL for individual i at time t provided by the growth curve and it is computed by subtracting the observed mean and dividing by the observed standard deviation. The coefficient *α*_*s*_ is the intercept, *β*_*s1*_ is the coefficient of sex and *β*_*s2*_ and *β*_*s3*_ are the coefficients of the linear and quadratic predictors of SVL. The quantities *β*_*s4*_ and *β*_*s5*_ are coefficients for the interaction terms, while $\epsilon_{t}^{\left(\phi \right)}$ is the random year effect for survival. The general model for recapture probability, *p*, was similar:

(5) $$\text{logit}\left(p_{i,t}\right)=\alpha_{p}+\beta_{p1}\cdot {\mathrm{sex}}_{i}+\beta_{p2}\cdot {\mathrm{SVL}}_{i,t}+\beta_{p3}\cdot {\mathrm{sex}}_{i}\cdot {\mathrm{SVL}}_{i,t}+\varepsilon_{t}^{\left(p\right)}.$$

The quadratic term to describe the relationship between body size and survival was considered to account for a possible increase in early life stages followed by a decrease in old lizards (see e.g. Massot et al., 2011). We did not consider a similar hypothesis for recapture probability (see Tenan et al., 2013). Sex was modelled as a latent variable to be estimated: sex_*𝑖*_ ∼ Bern(ψ), ψ is the population‐level sex ratio which was assigned a uniform prior on (0, 1). Model selection was performed with the Gibbs variable selection procedure (GVS; Dellaportas et al., 2000; Ntzoufras, 2002) to measure the support for the inclusion of a given predictor on survival and recapture. Pseudopriors for the parameters (*β*_*sr*_ and *β*_*pr*_) were first derived from a pilot run of the full model, with a mixture of a normal distribution and an inverse gamma hyperprior for the variance, *σ* ^*2*^_*𝑖*_ ∼ Γ^*−1*^(4, 5) (see Keevil et al., 2018; King et al., 2009). In the GVS procedure, each parameter of interest (*β*_*sr*_ or *β*_*pr*_) was multiplied by an inclusion variable, $g_{r}$, which was modelled with a Bernoulli prior distribution with parameter 0.5. On interactions, when $g_{r}=0\;$for one or both of the corresponding main effects, the product between the inclusion variable and the parameter of interest dropped out of the likelihood (Dellaportas et al., 2000). In order to improve mixing, posterior samples of the pseudoprior parameters were used in the GVS model (Dellaportas et al., 2000; Ntzoufras, 2002; Tenan et al., 2014), The mean value of $g_{r}$ in the MCMC output estimates the marginal posterior probability averaged across models that include the *r*‐th predictor (O'Hara & Sillanpää, 2009; Tenan et al., 2014). Combining survival and recapture sub‐models results in a total model space of 10 × 5 models and the GVS procedure was used to assess support for the inclusion of parameters affecting *ϕ* and *p*. The model proposed at each iteration of the MCMC chain was encoded as:

(6) $$\mathrm{mdl}=1+\sum_{r=1}^{P}2^{r}g_{r},$$

for *r* = 1:P inclusion parameters. Converting *mdl* back to binary notation reveals the status of each inclusion parameter and allows for calculation of the marginal posterior probability of sex and body size effects (Ntzoufras, 2002). We ran GVS model on five chains for 150,000 sampling iterations, with a burn‐in of 10,000 iterations. Inference was based on parameter estimates from a model that included predictors having a marginal inclusion probability >0.5 (Barbieri & Berger, 2004) and the model with the largest posterior probability was selected as the best model. Then, we ran the best model selected on five chains for 150,000 sampling iterations and a burn‐in of 50,000 iterations. We evaluated mixing and convergence of chains by inspecting trace plots and the *R̂* measures for all parameters (Brooks & Gelman, 1998). The integrated model was fit in the program JAGS (Plummer, 2003), accessed through R (version 4.0; R Core Team, 2019).

### 2.5 Life span

Assuming a constant survival (*ϕ*) after size x, the expected average life span, *l*_*x*_, from size x (Charnov & Berrigan, 1990; Seber, 1982) can be estimated as:

(7) $$l_{x}=-1/\ln\left(\varphi \right).$$

The average life span calculation took the uncertainty in survival estimates into consideration. We thus used posterior samples for survival probability and derived the 95% credible interval (95% CRI) of the expected life span. The parameters and data used for the integrated analysis are described in the supporting information (Appendix S1; Table S1.1) and the code to run the model is available online (see data availability statement).

### 2.6 Systematic review of growth and life span in *Podarcis* spp.

The aim of the systematic review was to gather data on growth, survival, sexual maturity and life span of insular and mainland populations of *Podarcis* species. We conducted a systematic literature review of the articles published up to 2022, using two databases (Scopus and PubMed) and other reports (e.g. Rotger Vallespir, 2016; Salvador, 2009). The exact search terms used can be found in the Appendix S2 (List S2), and the inclusion and exclusion criteria are described in Appendix S2 (Table S2.1). Systematic review retrieved 1755 records which were screened first by abstract, and then by full texts, both by two reviewers. The details on the systematic review can be found in PRISMA diagram in Appendix S2 (Figure S2). Altogether, we detected 20 studies that meet the eligibility criteria. However, studies in captivity were excluded in the comparison between insular and mainland populations.

When the same parameters of interest (body size, growth or survival) were reported in the same study separated by sex, we selected the maximum value to avoid discrepancies with other studies that used maximum population values. When only the range of age at first reproduction was reported we took the mean value. Values of *K* reported were referred to the growth coefficient which is the rate (1 year) at which the asymptotic length is approached. It is not a growth rate and it can be view as the ‘fixed fraction by which the annual growth increment is multiplied each year’ (Schnute & Fournier, 1980). To test if *K*, life span and age at first reproduction were different between mainland and insular populations of *Podarcis* species, we used Wilcoxon's sum rank tests.

## 3 RESULTS

### 3.1 Body size growth analysis

**FIGURE 1:**
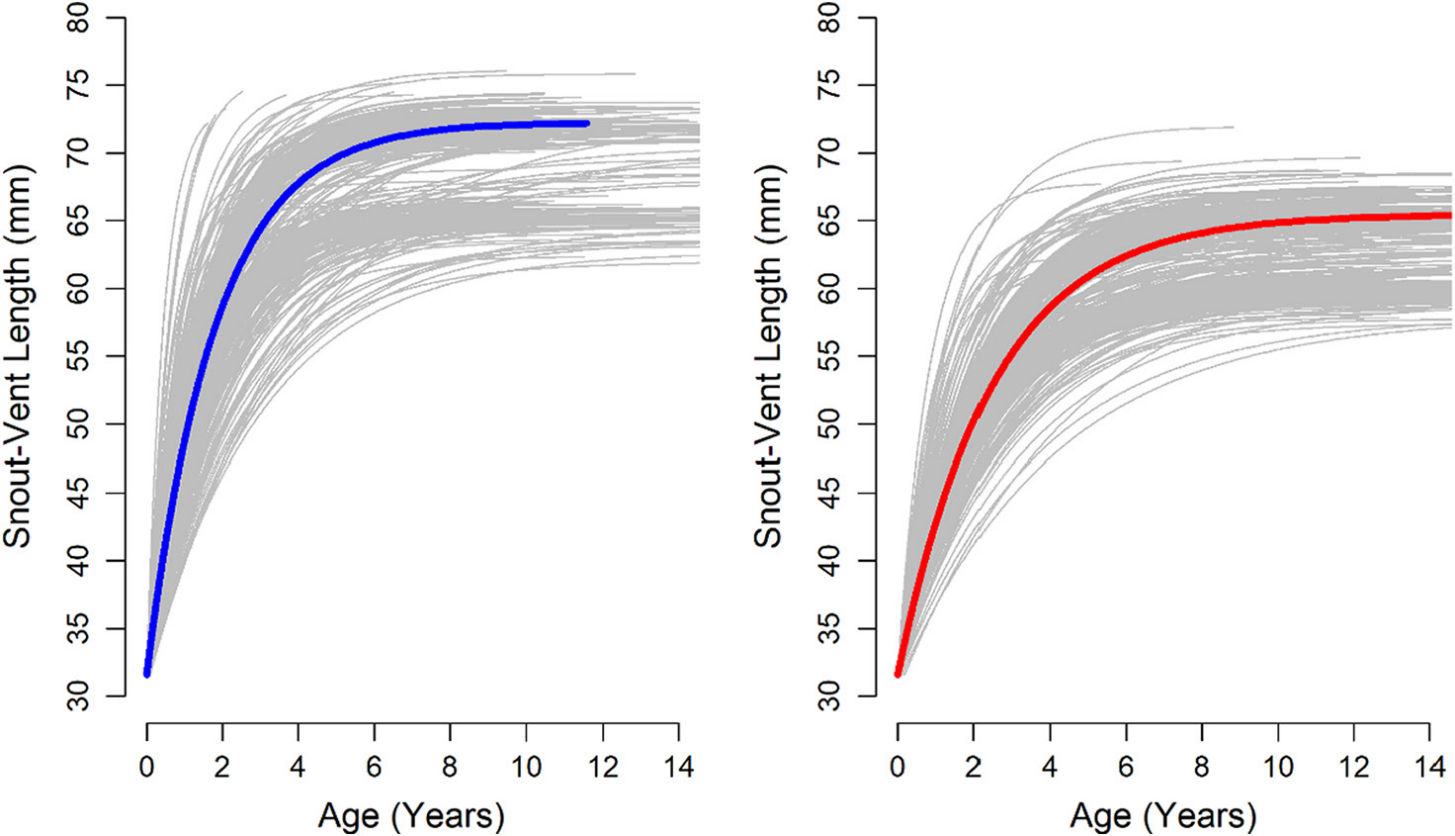
Growth curves for male (left panel) and female (right panel) Lilford's lizard. Grey curves display the individual growth curves; coloured lines are the average growth curve for both sexes.

We gathered 1650 captures of 730 individuals (332 males, 385 females and 13 for which sex was unknown at capture and was later assigned by the model) with body size between 36 and 78 mm. Both sexes had a small averaged growth coefficient (*K*), but males had a faster growth than females (Table 1) and attained larger sizes. Derived growth curves showed that SVL increased almost linearly between hatching and sexual maturity for both sexes, while the growth rate abated between age 5 and 6 (Figure 1). Sexual maturity is estimated to occur at age 2 for both sexes (after rounding, Table 1), but all main parameters (*K* and *L*_*T2*_) showed a large interindividual variability (Appendix S1; Table S1.2).

**TABLE 1:** Growth parameters estimates of the Schnute growth model

	L∞ (mm)	*K* (year^−1^)	Age at maturity (year)
Male	72.3 (71.2–73.2)	0.56 (0.48–0.65)	2.00 (1.42–2.75)
Female	65.6 (65.1–66.0)	0.41 (0.34–0.49)	1.91 (1.28–3.01)

L∞ = average asymptotic body size attained by lizards, *K* = average growth coefficient. The 95% credible intervals are in brackets

### 3.2 Hierarchical models for survival analysis and average life span

**FIGURE 2:**
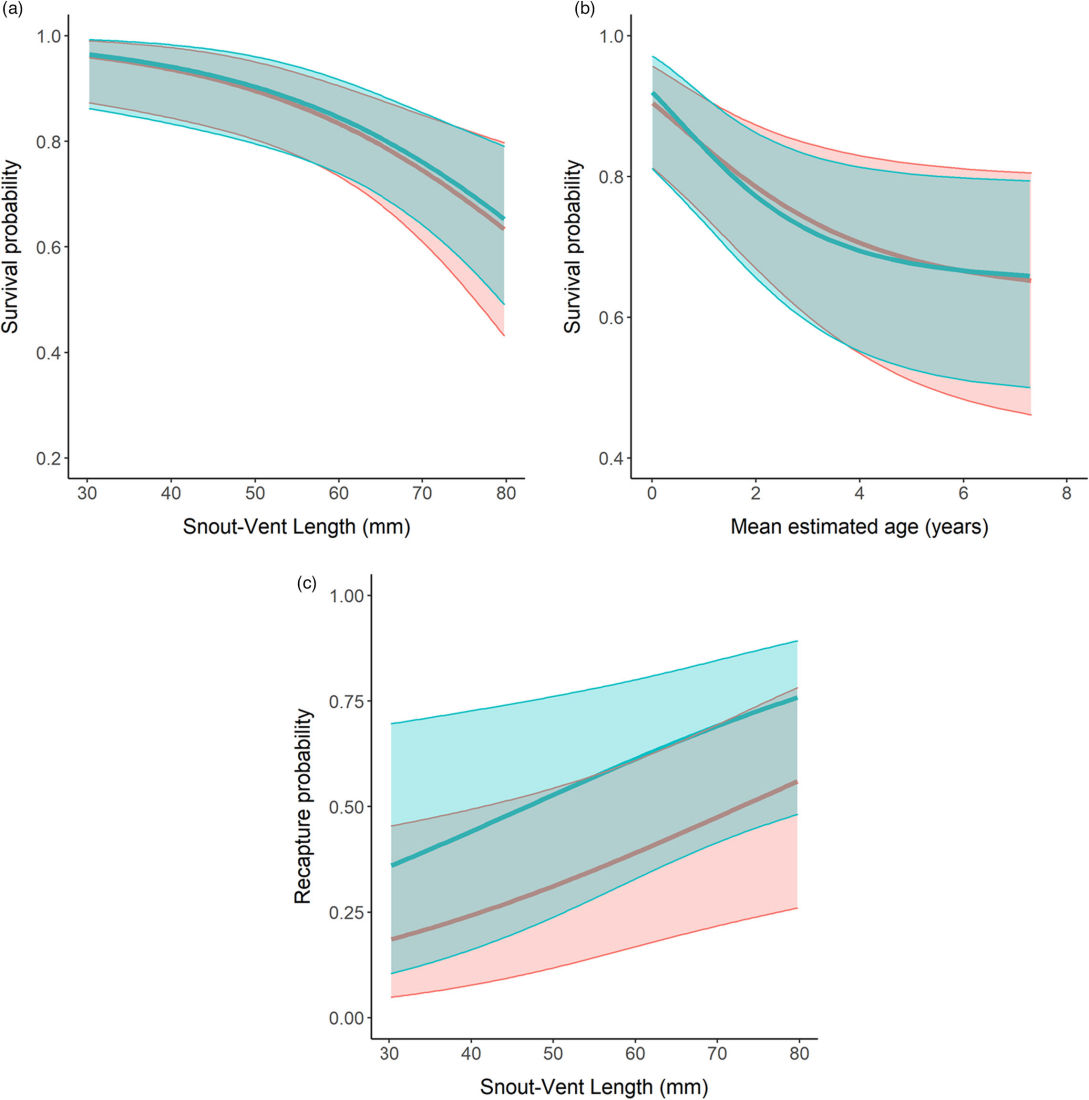
(a) Size‐dependent survival probabilities of *Podarcis lilfordi* from the model with the greatest support. (b) Age‐dependent survival probability using age‐by‐size transformation (Equation 3 in the text). (c) Size‐dependent recapture probability of males and females. Blue line = males, red line = females. Shaded areas indicate the 95% credible interval.

**FIGURE 3:**
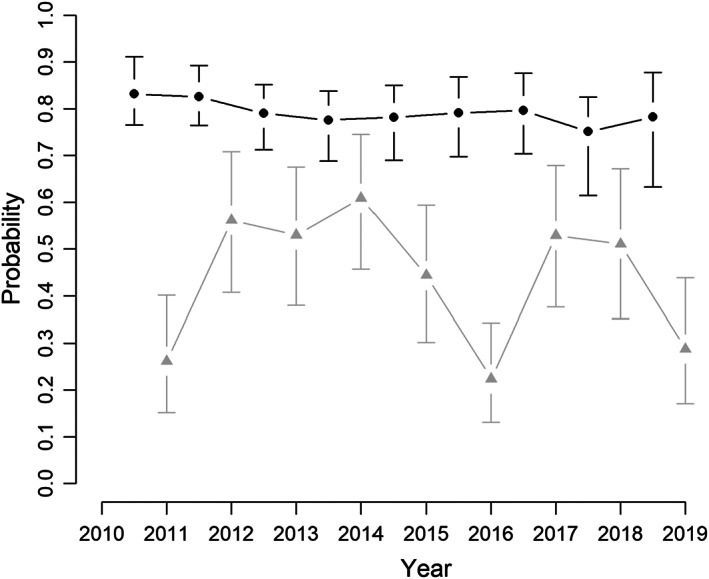
Average annual survival (black) and recapture (grey) probabilities. Points represent posterior mean values and lines are the 95% credible intervals.

**FIGURE 4:**
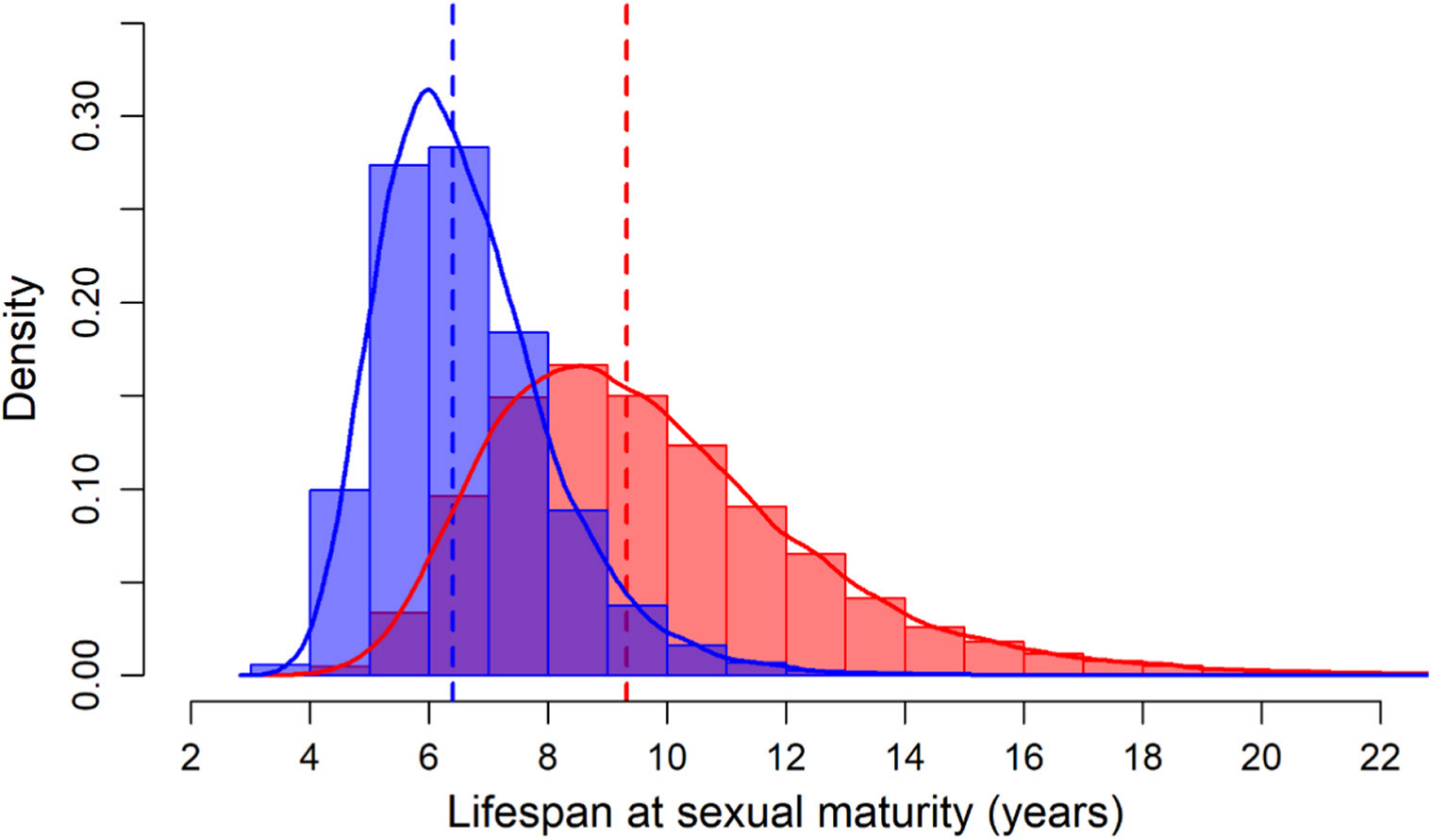
Histogram of posterior distribution of mean life expectancy in males (blue) and females (red) after reaching sexual maturity (~2 years). Dashed lines indicate the median values of each sex.

The model including the effects of sex and body size as predictors of both survival and recapture probability achieved >30% posterior probability following the GVS procedure, and it was selected as the best model (Table 2). The interaction term between the effect of sex and body size was supported in about half of the iterations (45%), followed by the quadratic effect of size (19%; Table 2). Annual survival probability of the smallest individuals was high (~0.95) and progressively abates with lizard size (Figure 2a). However, the effect of sex on the survival probability was close to zero (i.e. it is 95% CRI encompasses zero; Appendix S1; Figure S1.1), and no visual differences were found in survival between sexes (Figure 2b). The difference between the age‐dependent curve and the size‐dependent curve was due to the faster growth of males during the first year of life (Figure 2b). There was strong support for the additive effect of sex (100%) and body size (80%) in the capture probability (Table 2), with the highest capture probability for large males (Figure 2c). Annual survival varied little between years, ranging on average from 0.75 (95% CRI: 0.61–0.83) in 2018 to 0.83 (95% CRI: 0.76–0.91) in 2011. Otherwise, annual recapture probability was much more variable ranging on average from 0.22 (95% CRI: 0.17–0.30) in 2016 to 0.61 (95% CRI: 0.52–0.69) in 2014 (Figure 3). The standard deviation for the temporal random effect on survival was about half that as of for recapture (Appendix S1, Table S1.2). The average annual survival probability was high and similar in both sexes (0.79; 95% CRI: 0.73–0.84 and 0.81; 95% CRI: 0.68–0.89, for females and males respectively). The oldest male observed in our population was captured in 2010 as an adult (SVL = 70 mm) and was recaptured 10 years later at an estimated age of ≥13 years old. Similarly, the oldest female in the dataset was captured as an adult lizard in 2011 (SVL = 64 mm) and was recaptured in 2019 (SVL = 65 mm) at an estimated age of ≥12 years old. Following Equation 7 the median life span after reaching the size at maturity (59 mm and 50 mm in males and females respectively) is 6.18 (95% CRI: 3.68–11.42) years for males and 8.99 (95% CRI: 4.91–18.15) years for females (Figure 4).

**TABLE 2:** Posterior probability of the best models (posterior probability ≥ 0.05) among the 50 models tested

Model notation	Survival probability					Recapture probability			Posterior model probability
	*β*_s_(sex)	*β*_s_(SVL)	*β*_s_(SVL2)	*β*_s_(sex*SVL)	*β*_s_(sex*SVL2)	*β*_p_(sex)	*β*_p_(SVL)	*β*_p_(sex*SVL)	
φ(sex + svl), p(sex + svl)	1	1	‐	‐	‐	1	1	‐	0.312
φ(sex * svl), p(sex + svl)	1	1	‐	1	‐	1	1	‐	0.209
φ(sex + svl), p(sex * svl)	1	1	‐	1	‐	1	1	1	0.084
φ(sex * svl), p(sex)	1	1	‐	1	‐	1	‐	‐	0.084
φ(sex + svl), p(sex)	1	1	‐	‐	‐	1	‐	‐	0.074
φ(sex * svl), p(sex * svl)	1	1	‐	1	‐	1	1	1	0.051
φ(sex + svl + svl2), p(sex + svl)	1	1	1	‐	‐	1	1	‐	0.050
**Pr(Included)**	1	1	0.186	0.447	0.035	1	0.804	0.164	

The bottom row shows the posterior probability of individual covariates being included in the model. This probability is based on the number of model iterations in which that given covariate was included (see text for details). Notation: *β*_s_ = linear predictor of the covariate/effect on survival, *β*_p_ = linear predictor of the covariate/effect on recapture. SVL = snout‐to‐vent length, SVL2 = quadratic term of snout‐to‐vent length, ‐ = predictor not included into the model

### 3.3 Systematic review of growth and life span in *Podarcis* spp.

**FIGURE 5:**
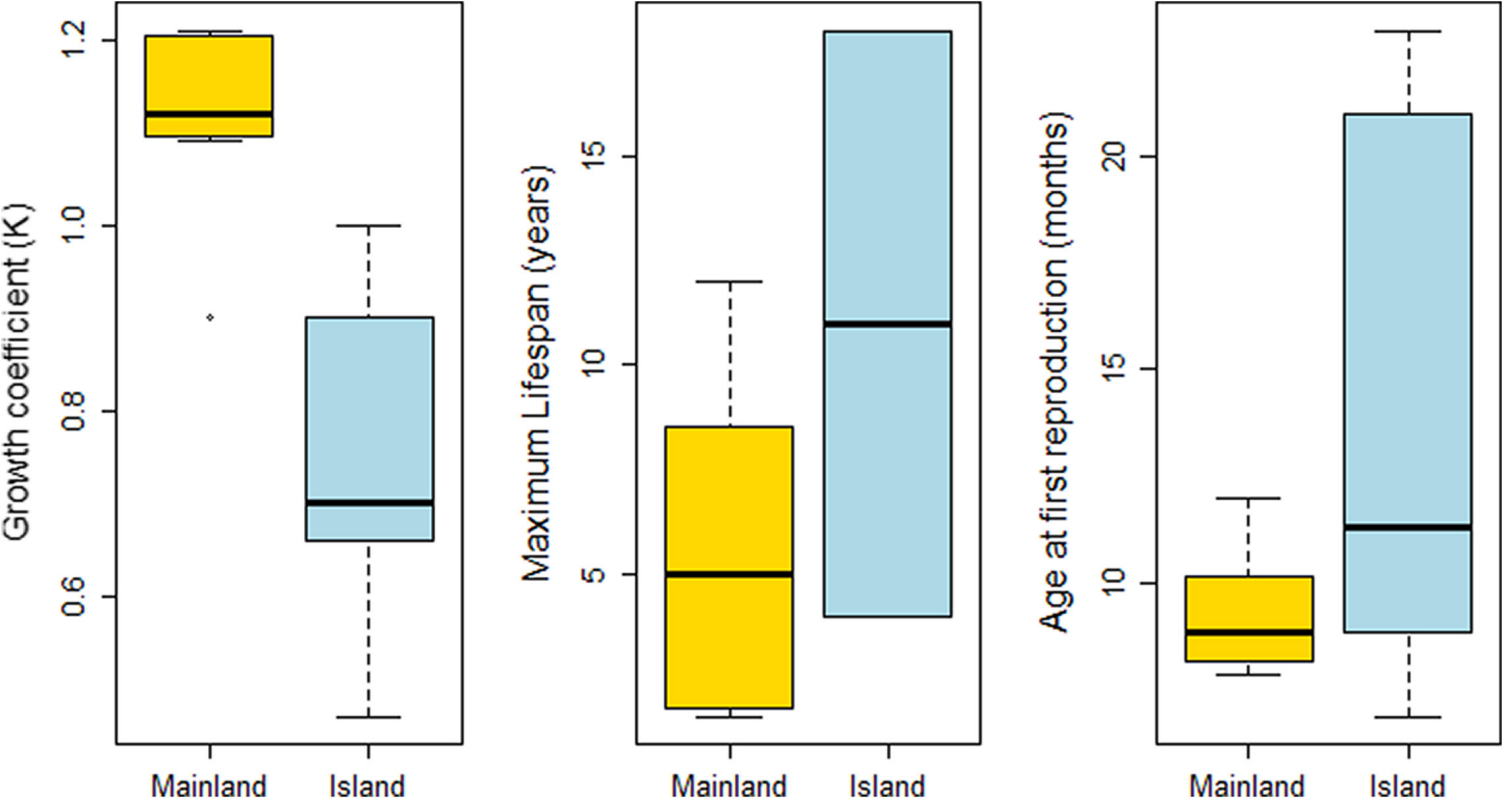
Boxplots comparing the growth coefficient, *K*, of insular (light blue) and mainland (yellow) populations of *Podarcis* species (left panel), the maximum life span calculated in years of insular and mainland populations of *Podarcis* species (middle panel), and the age at first reproduction in months (right panel).

Our systematic review identified 20 studies that provided data on 29 populations (14 island, 15 mainland) of 16 *Podarcis* species (Appendix S2; Figure S2 and Table S2.2). Growth coefficient (*K*) between lizards on islands was lower than on the mainland (Mean ± SD; Island: 0.73 ± 0.18, Mainland: 1.12 ± 0.10) being statistically significant (Wilcoxon sum rank test, *p* = 0.001). Our study population had values below the average of insular populations (0.41 ± 0.10 and 0.56 ± 0.12 in females and males respectively). Maximum life span of Lilford's wall lizard populations varied between 13.9 years in captivity (Scharf et al., 2015) to more than 18 years in this study population. Maximum life span in years was largely higher on islands on average (Mean ± SD; Island: 11.0 ± 8.08, Mainland: 5.6 ± 4.23), however, not reaching statistical significance (Wilcoxon sum rank test, *p* = 0.3). This was probably due to the high variability of this parameter among insular populations (Figure 5). Otherwise, individuals on islands mature later than individuals from mainland. The age at first reproduction in months varied from 7 to 21 months in the reported populations (Mean ± SD; Island: 13.73 ± 6.80, Mainland: 9.36 ± 1.51) being statistically significant (Wilcoxon sum rank test, *p* = 0.003; Figure 5). In our study population, age at first reproduction was estimated around of 24 months considerably higher than the average of other insular populations of *Podarcis* species.

## 4 DISCUSSION

We found that Lilford's wall lizard in this island population had a higher survival and a greater maximum life expectancy than other mainland species as predicted by the island syndrome (Adler & Levins, 1994; Andrews, 1976; McNab, 2002; Roff, 2002). These traits are probably an adaptation to limited food resources in this small islet. Per‐capita food resources is a critical factor that modulates the somatic growth rate in reptiles (Andrews, 1982; Heino & Kaitala, 1999). In lizards, fast growth is promoted by intraspecific competition and high food availability (Dickinson & Fa, 2000; Pafilis et al., 2009), whereas food limitation results in a ‘slow’ life‐history tactic (McNab, 2002, but see Raia et al., 2010). The comparison between island and mainland populations across 16 *Podarcis* species further supports the slow growth and late age of sexual maturity of insular populations (Figure 5). Although the maximum life span of insular populations was not statistically significant, there are great population differences within the insular groups, indicating that island specific selective pressures or random genetic processes might play contrasting roles (Rotger et al., 2020, 2021). Rotger et al. (2020) compared the growth of rate of female Lilford's lizard from three insular and neighbouring populations, including the one studied here. They found that the growth rate of females was modulated by food availability. We extended this model to males and a continuous growth function with a survival model to provide detailed results on sex‐ and size‐dependent tactics. We showed that males had a higher growth coefficient (K) and reach a larger asymptotic body size ($L_{\infty }$) than females, as observed in other *Podarcis* species (Adamopoulou et al., 2000; Galán, 1999). When comparing age with size at maturity, males reached sexual maturity at a similar age, but matured at a larger size than females. The faster growth of males might be an indication of intrasexual despotic competition for food and mating (Mugabo et al., 2010; Pérez‐Mellado et al., 2015). Raia et al. (2010) reported that despotic competition drives male strategy when resource availability is stochastic. Another important result is the large variability between individuals in both the asymptotic size and the growth coefficient (Figure 1, Appendix S1; Figure S1.2, see also King et al., 2016, Rotger et al., 2016). This variability is likely to reflect hatching date (spring vs. autumn) and differences in the initial conditions of early life (Massot & Aragón, 2013; Olsson & Shine, 2002). Rotger et al. (2016) showed that the growth rate of newborn Lilford's lizards is determined by climatic condition and per‐capita food resources. Similarly, Mugabo et al. (2010) experimentally showed a positive relation between food intake early in life and growth rate in the common lizard, *Zootoca vivipara*.

Together with slow growth, lizards in our population lived long (average annual survival probability ~0.80). When projected across the lizard's life, the size‐dependent survival found here suggests that about 9% of newborn lizards reach the age of 10. Indeed, among the 129 lizards released during the first year of the study, 20% were still alive after 6 years. To our knowledge, the estimate found here is the highest value of annual survival ever registered in the wild for a *Podarcis* species (Appendix S2; Table S2.2). Comparing survival across *Podarcis* species is difficult because most studies report the maximum observed life span, *l*_max_., which refer to a single individual, often kept in captive conditions. Nevertheless, Benítez‐López et al. (2021) reported that *l*_max_ in insular populations is generally higher than in mainland. The high survival rate, especially in newborn lizards, is predicted by the island syndrome and it is likely the consequence of a reduced predator pressure and interspecific competition (Schoener & Schoener, 1982). A consequence of the high survival is the high population density found in Lilford's lizard populations (Pérez‐Mellado et al., 2008) and the low fecundity of the species (Castilla & Bauwens, 2000). In our dataset, newborn lizards (SVL < 45 mm) represent only 1% of the individuals (8/730). Even accounting for a low detection probability, this small number is an indication that fecundity is low. A low fecundity is typical of food limited populations and is predicted by the island syndrome (Jordan et al., 2005; Novosolov et al., 2013; Siliceo & Díaz, 2010).

A novel aspect of our study was the description of a survival pattern in which survival abates with the size of the lizard. Senescence patterns in small lizards are not well described and are even less understood in wild populations (Nussey et al., 2008, but see Patnaik, 1994). Compared with mammals and birds (Owen et al., 2008), ageing in reptiles is much slower (e.g. Tully et al., 2020; Warner et al., 2016). Warner et al. (2016) suggested that this is because the fitness benefits at large sizes may overcome the ‘declining power of natural selection with advancing age’. da Silva et al. (2022) reported the existence of a slight, nearly negligible, senescence among different species of testudines in captivity. We found that ageing of Lilford's lizard in our population is similar to this pattern, described in large reptiles (da Silva et al., 2022; Finch, 1998; Patnaik, 1994; Vaupel et al., 2004; Warner et al., 2016). Lilford's lizard survival pattern is in agreement with the prediction that senescence is evident in populations with low extrinsic mortality (Jordana et al., 2012; Reznick et al., 2004), indicating possible physiological mechanisms, that is, accumulation of altered enzyme molecules or a decrease in the responses to stress‐enhanced anti‐oxidative defence mechanisms (Patnaik, 1994). However, evidence suggests that site‐specific selective pressures can generate contrasting life‐history tactics across population of the same species (Rotger et al., 2020, 2021).

## 5 CONCLUSIONS

Lizards in our study population have slow growth and a high average survival probability that decreased throughout the lizards' life span, as predicted by the island syndrome. However, other traits might not follow this rule, at least for *Podarcis* species. For example, maximum body size measures do not seem to have a clear pattern (Appendix S2; Table S2.2). In fact, Meiri et al. (2007) found that insular lizards are not larger than mainland counterparts, in contrast with the island syndrome predictions. Raia et al. (2010) also questioned that the island syndrome holds for the Italian wall lizard, *Podarcis sicula*, at Licosa island (Southern Italy), where the environmental unpredictability selects for a ‘fast’ strategy and animals are more aggressive and allocate more energy into reproduction than in mainland populations. In the same way, Novosolov et al. (2013) found that reproductive output in insular populations is comparable to that in the mainland populations because, although clutches on islands tend to be smaller, their frequency is higher than in mainland populations. However, island characteristics may play a more important role than isolation itself. For example, the high survival probability found here is also higher than the average survival probability in two other populations of Lilford's wall lizard (0.60 and 0.63, Rotger et al., 2020). A general conclusion on whether the island syndrome holds for insular lizard populations is difficult to draw. Traits are linked by multiple evolutionary trade‐offs, and it is likely that multiple strategies can evolve in different islands. Across its distribution, *P. lilfordi* exhibits a large morphological diversity in colour, body size and sexual dimorphism (Rotger et al., 2016; Salvador, 1980). Rotger et al. (2020) found that body size differences across three neighbouring populations ultimately resulted in different life‐history tactics due to evolutionary trade‐offs. Here, we illustrated how to estimate survival in relation to a continuous trait like body size and we hope this can foster future studies on the role of body size in shaping insular traits especially in species with continuous growth.

## AUTHOR CONTRIBUTIONS

Giacomo Tavecchia, José‐Manuel Igual and Andreu Rotger conceived the idea; Simone Tenan, Simon Bonner and Andreu Rotger designed the methods; Giacomo Tavecchia, José‐Manuel Igual and Andreu Rotger performed fieldwork and collected the data; Giacomo Tavecchia and Andreu Rotger wrote the paper, José‐Manuel Igual, Simon Bonner and Simone Tenan reviewed the paper; Andreu Rotger analysed the data; Giacomo Tavecchia contributed with logistics, and all authors contributed to editing the final manuscript.

## CONFLICT OF INTEREST

The authors have no conflict of interest.

## Supporting information


Appendix S1.

Appendix S2.

## Data Availability

R Codes and data to conduct analysis are available on Figshare https://doi.org/10.6084/m9.figshare.17060300.v2 (Rotger et al., 2022).
