# Supplementary material for: Life span, growth, senescence and island syndrome: Accounting for imperfect detection and continuous growth
Source: J Anim Ecol. 2022 Nov 17;92(1):183–94. doi: 10.1111/1365-2656.13842 (PMC10099801; doi:10.1111/1365-2656.13842)

**Supporting Information.** Rotger, A., S. Tenan, J.M. Igual, S. Bonner and G. Tavecchia. 2021. Lifespan, growth, senescence and island syndrome: accounting for imperfect detection and continuous growth.

**Appendix S1**

**Table S1.1.** Notation and biological meaning of data and of parameters of interest.

**Table S1.2.** Growth and CJS model parameters, priors, and estimates from the best model.

**Figure S1.1.** Effcts of sex and body size (SVL) on survival and recapture probability. Points represent posterior means. Thick and thin lines represent 50% and 95% credible intervals, respectively.

**Figure S1.2.** Traceplots and posterior distributions of growth parameters used to check possible bimodality. Asym = asymptoptic size, K= growth coefficient.

**Table S1.1**

| **Growth parameters** |  |
| --- | --- |
| *Hs_i_* | Hatchling body size of individual *i* in mm |
| *y2_i_* | The average body size at the maximum age of the individual *i* in mm |
| *K_i_* | Growth coefficient of the individual *i* (1/years) |
| **Demographic parameters** |  |
| *phi_i,t_* | Survival probability of individual *i* in year *t* |
| *p_i,t_* | Recapture probability of individual *i* in year *t* |
| **Observational data** |  |
| *y_i,1:T_* | Capture history of individual i over from the first (1) to the last (T) year of the study period |
| *Lr_i,1:T_* | Body size capture history in mm over from the first (1) to the last (T) year of the study period |

**Table S1.2**

|  | **Parameters** | **Prior distribution** | **Prior values** | **Mean** | **SD** | **2.50%** | **97.50%** |
| --- | --- | --- | --- | --- | --- | --- | --- |
| **Growth** | Mean Hatchling SVL (H_SVL_) | Uniform | (25, 35) | 31.86 | 2.51 | 25.96 | 34.91 |
|  | SD, individual random effect on Hs (σ_Hsvl_) | Uniform | (2, 3) | 2.50 | 0.29 | 2.02 | 2.98 |
|  | Maximun SVL at time T_2_ (y_2_) | Uniform | (65, 70) | 65.47 | 0.21 | 65.08 | 65.90 |
|  | Sex effect on y2 (male effect) | Uniform | (0, 15) | 6.73 | 0.29 | 6.11 | 7.29 |
|  | SD, individual random effect on y2 (σ_y2_) | Uniform | (0, 5) | 1.80 | 0.15 | 1.51 | 2.11 |
|  | Growth coefficient (log(K)) | Uniform | (-8,-5) | -6.79 | 0.10 | -6.99 | -6.60 |
|  | Sex effect on log(K) (male effect) | Uniform | (0,1.5) | 0.31 | 0.12 | 0.08 | 0.55 |
|  | SD, individual random effect on K (σ_K_) | Uniform | (0,2) | 0.61 | 0.06 | 0.49 | 0.73 |
|  | SD, individual random effect on size (σ_SVL_) | Uniform | (0,5) | 1.19 | 0.04 | 1.12 | 1.27 |
| **Survival** | Mean survival (φ) | Uniform | (0,1) | 0.79 | 0.03 | 0.73 | 0.84 |
|  | Sex effect on φ (β_sex_) | Uniform | (-5,5) | 0.09 | 0.17 | -0.25 | 0.43 |
|  | Linear size effect on φ (β_SVL_) | Uniform | (-5,5) | -0.34 | 0.12 | -0.58 | -0.12 |
|  | SD, time random effect on φ (σ_φ-t_) | Uniform | (0,5) | 0.30 | 0.20 | 0.02 | 0.78 |
| **Capture** | Mean capture probability (p) | Uniform | (0,1) | 0.44 | 0.07 | 0.30 | 0.58 |
|  | Sex effect on p (β_sex_) | Uniform | (-5,5) | 0.91 | 0.15 | 0.61 | 1.21 |
|  | Linear size effect on p (β_SVL_) | Uniform | (-5,5) | 0.22 | 0.08 | 0.06 | 0.38 |
|  | SD, time random effect on p (σ_p-t_) | Uniform | (0,5) | 0.78 | 0.26 | 0.43 | 1.43 |

**Figure S1.1**


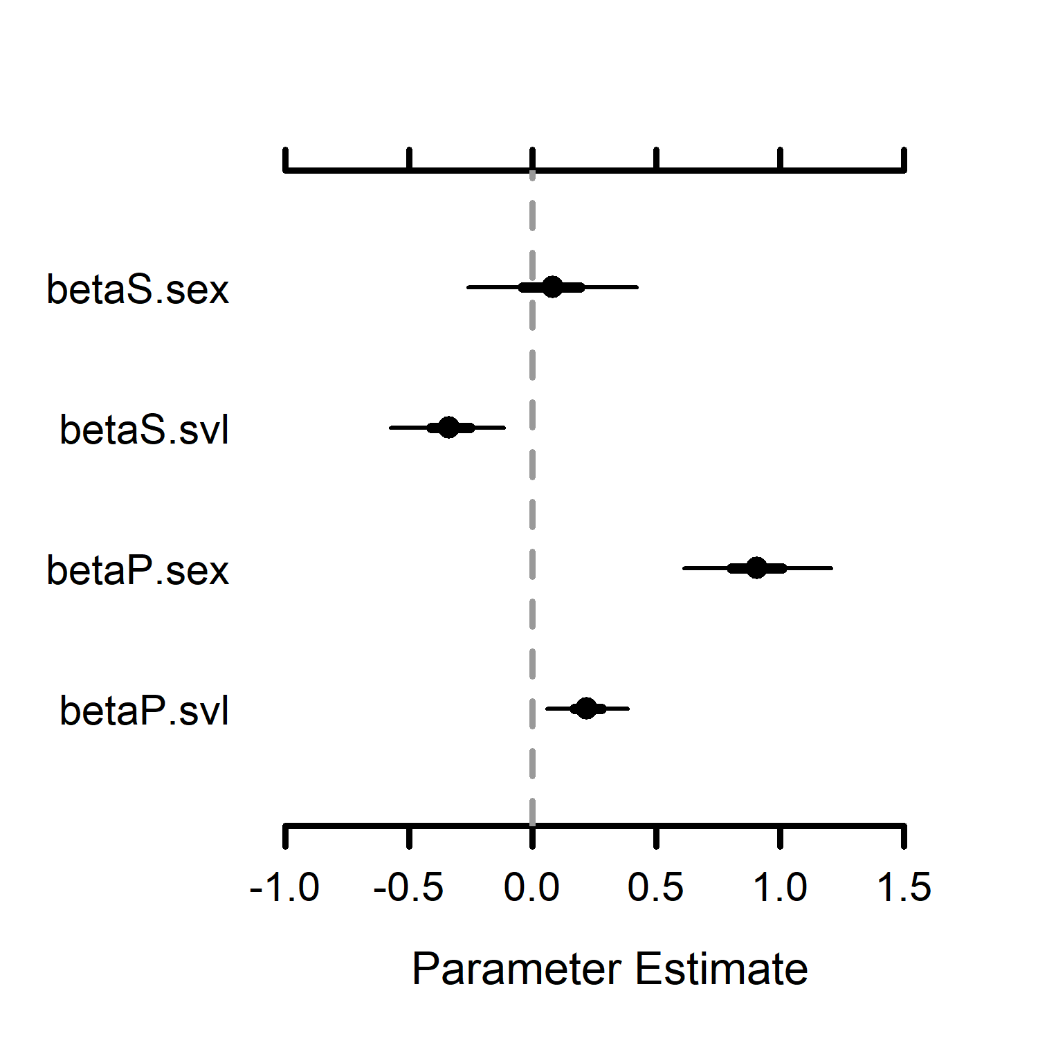


**Figure S1.2**


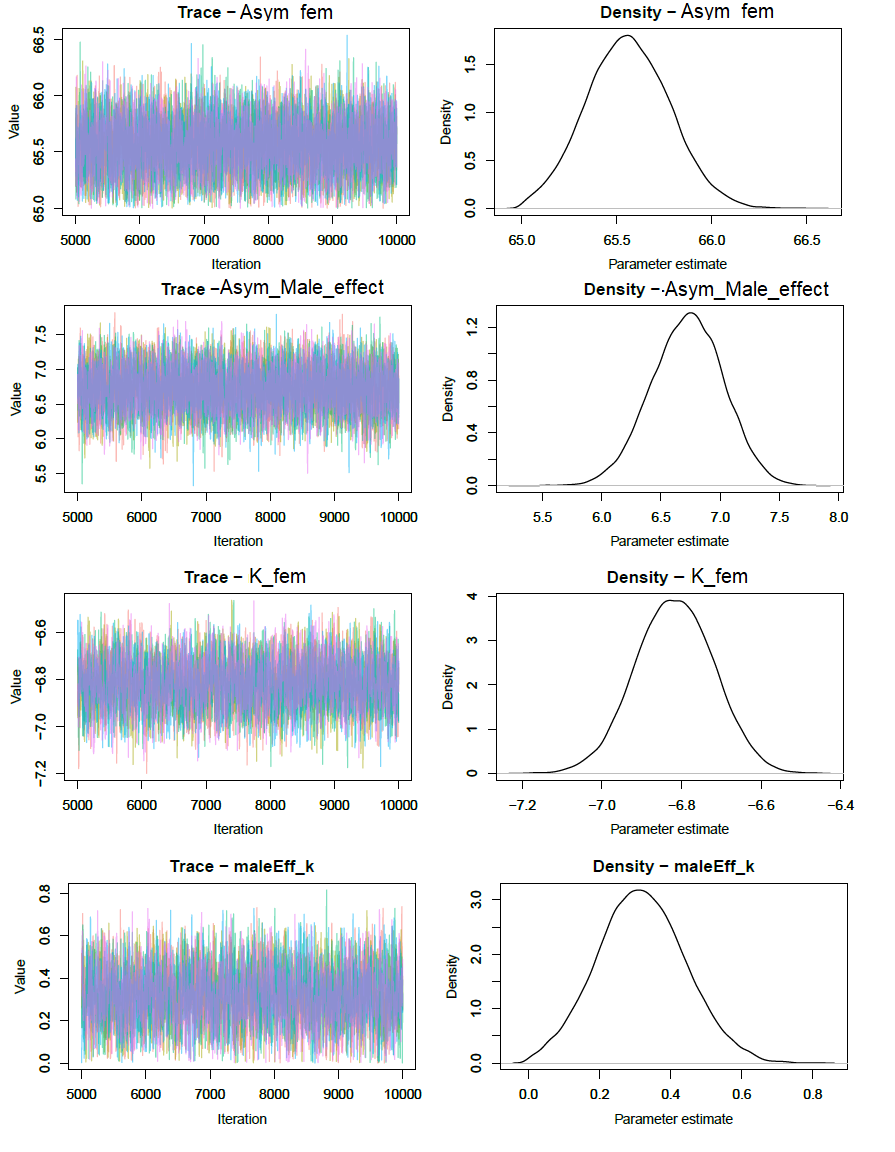

Supplement: Supplementary file 1 — Appendix S1. [file JANE-92-183-s001.docx]
