# Supplementary material for: Life span, growth, senescence and island syndrome: Accounting for imperfect detection and continuous growth
Source: J Anim Ecol. 2022 Nov 17;92(1):183–94. doi: 10.1111/1365-2656.13842 (PMC10099801; doi:10.1111/1365-2656.13842)
Supplement: Supplementary file 2 — Appendix S2. [file JANE-92-183-s002.docx]

**Supporting Information.** Rotger, A., S. Tenan, J.M. Igual, S. Bonner and G. Tavecchia. 2021. Lifespan, growth, senescence and island syndrome: accounting for imperfect detection and continuous growth.

**Appendix S2**

**List S2.** Search terms used in Pubmed and Scopus database.

**Table S2.1.** Inclusion/exclusion criteria used in the screening process of the systematic review.

**Figure S2.** PRISMA flow diagram of the literature searching and selection. Note that numbers of articles or reports are reported at each stage.

**Table S2.2.** Ecological parameters in mainland and insular populations of *Podarcis* species. L_max_ is the maximum body size (SVL) in mm, T_mat_ is the age at first reproduction in months, T_max_ the maximum lifespan in years, S_max_ is the corresponding survival that was calculated as: exp(-1/ T_max_), and survival rate (S_r_) was the survival obtained from CR studies, and growth coefficient (*K*) was calculated from the Von Bertalanffy or Schnute equation.

**List S2**

**Pubmed May-2022 (general search = 693 records until 2021)**

(lizard[Title/Abstract]) AND (diversification[Title/Abstract] OR island[Title/Abstract] OR insular[Title/Abstract] OR study[Title/Abstract] OR comparative[Title/Abstract]) AND (reproductive[Title/Abstract] OR body size[Title/Abstract] OR growth[Title/Abstract] OR life history[Title/Abstract] OR survival[Title/Abstract] OR sexual[Title/Abstract])

**SCOPUS May-2022 (general search = 1075 records)**

TITLE-ABS-KEY("lacertidae" OR "lizard*" OR "reptil*")AND TITLE-ABS-KEY("diversification" OR "Island*" OR "worldwide" )AND TITLE-ABS-KEY("body size" OR "survival" OR "growth" OR "longevity" OR "age" OR "sex")AND PUBYEAR > 1949
AND PUBYEAR < 2022
AND (
LIMIT-TO ( SUBJAREA,"AGRI" )
OR LIMIT-TO ( SUBJAREA,"ENVI" )
)
AND (
LIMIT-TO ( DOCTYPE,"ar" )
OR LIMIT-TO ( DOCTYPE,"re" )
)

AND (
LIMIT-TO ( LANGUAGE,"English" )
OR LIMIT-TO ( LANGUAGE,"Spanish" )
OR LIMIT-TO ( LANGUAGE,"Catalan" )
)

**SCOPUS May-2022 (target search to study species = 234 records)**

(TITLE-ABS-KEY("podarcis")AND TITLE-ABS-KEY("study" OR "insular" OR "syndrome" OR "islan*" OR "rule" OR "mainland" OR "population*" OR "mediterranean")AND TITLE-ABS-KEY("survival" OR "growth" OR "rate" OR "age" OR "sex*" OR "longevity" OR "trait" OR "mortality" OR "body size*" OR "morpholog*" OR "reproduct*" OR "offspring")AND TITLE-ABS-KEY("ecology" OR "evolution" OR "skeletochronology" OR "demograph*" OR "characteristic*" OR "Lacertidae"))
AND PUBYEAR < 2022
AND (
LIMIT-TO ( PUBSTAGE,"final" )
)
AND (
LIMIT-TO ( DOCTYPE,"ar" )
OR LIMIT-TO ( DOCTYPE,"re" )
)
AND (
LIMIT-TO ( SUBJAREA,"AGRI" )
OR LIMIT-TO ( SUBJAREA,"ENVI" )
)
AND (
LIMIT-TO ( LANGUAGE,"English" )
OR LIMIT-TO ( LANGUAGE,"Spanish" )
OR LIMIT-TO ( LANGUAGE,"Catalan" )
)

**Table S2.1**

| **Exclusion criteria** | |
| --- | --- |
| 1. | Manuscripts were not written in English, Spanish or Catalan. |
| 2. | Manuscripts only included information about lizard species that were not *Podarcis* (if that information was not clear in the abstract, the document was left for the following round). |
| 3. | Manuscripts studied urban or invasive populations of *Podarcis* species |
| 4. | Manuscripts studied behaviour, genetics or historical trends of the *Podarcis* species. |
| **Inclusion criteria** | |
| 1. | Manuscripts were a review or meta-analysis about lizard species and/or populations or manuscripts that compared different lizard species that *Podarcis* species were included. |
| 2. | Manuscripts studied demography, morphology, ecology or evolution of lizard species that included *Podarcis* species. |
| 3. | Manuscripts studied the somatic growth, skeletochronology or growth curves of lizard populations that included *Podarcis* species. |

**Figure S2.** PRISMA flow diagram of the literature searching and selection. Note that numbers of articles or reports are reported at each stage.

*Many information taken from public and technical reports published in: http://www.vertebradosibericos.org/reptiles.html and https://podarcis.de/AS/Taxon.php?Genus=19

**Most of the records were excluded in phase I automatically by Rayyan using the following excluding keywords: “behaviour”, “genetic*”, “genom*”, “phylogeny”, “parasite”, “gut”, and “invasive”. Besides other duplicates records identified during the visual inspection.

**Table S2.2**

| **Species** | **Insular** | **Locality** | **food availability** | **presencence of predators** | **intraspecific competition** | **Lmax** | **Tmat** | **Tmax** | **Smax** | **Sr** | **K** | **source** |
| --- | --- | --- | --- | --- | --- | --- | --- | --- | --- | --- | --- | --- |
| Podarcis bocagei | no | Vigo (Spain) | high | high | high | 70 | 16 | 4.0 | 0.8 | 0.8 | - | 1, 2, 3, 4 |
| Podarcis bocagei | no | A Coruña (Spain) | high | high | high | 64 | 8 | 2.0 | 0.7 | - | 1.2 | 5 |
| Podarcis carbonelli | yes | Berlenga island (Portugal) | low | low | low | 70 | - | 6.0 | 0.9 | - | - | 6 |
| Podarcis erhardii | no | - | - | - | - | 71 | 11 | 5.0 | 0.8 | - | 1.1 | 5, 7 |
| Podarcis filfolensis | yes | Captivity | - | - | - | 86 | - | 2.0 | 0.6 | - | - | 4, 7 |
| Podarcis gaigeae | yes | Skyros island (Greece) | high | high | high | 85 | 11 | 5.0 | 0.8 | - | - | 4, 7 |
| Podarcis hispanica | no | Captivity | - | - | - | 74 | 8 | 13.0 | 0.9 | - | - | 7 |
| Podarcis hispanica | no | Asturias (Spain) | high | high | high | 57 | 8 | 1.6 | 0.5 | - | 1.2 | 4, 5 |
| Podarcis hispanica | no | Salamanca (Spain) | high | high | high | 68 | 8 | 1.6 | - | - | 1.1 | 7 |
| Podarcis hispanica | yes | Cies Island (Spain) | high | high | high | 68 | 7 | - | - | - | 0.9 | 5, 8 |
| Podarcis lilfordi | yes | Captivity | - | - | - | 81 | 12 | 13.9 | 0.9 | - | - | 4, 7, 9 |
| Podarcis lilfordi | yes | Es Curt (Spain) | low | low | low | 73* | 23 | 18.0 | 1.0 | 0.8 | 0.5 | This study |
| Podarcis lilfordi | yes | Moltona (Spain) | high | low | high | 75* | - | - | - | 0.6 | 0.7 | 10 |
| Podarcis lilfordi | yes | NaGuardia (Spain) | high | low | high | 68* | - | - | - | 0.5 | 0.7 | 10 |
| Podarcis lilfordi | yes | Cabrera (Spain) | high | low | high | 70 | - | - | - | - | 0.7 | 9 |
| Podarcis liolepis | yes | Columbrets island (Spain) | high | high | high | 71 | 9 | 4.0 | 0.8 | - | 1.0 | 4, 5, 11 |
| Podarcis melisellensis | no | Captivity | - | - | - | 74 | - | 3.9 | 0.8 | - | - | 4, 7 |
| Podarcis milensis | yes | Milos island (Greece) | low | high | low | 63 | 14 | 4.0 | 0.8 | - | 0.5 | 12, 13 |
| Podarcis muralis | no | Captivity | - | - | - | 80 | 21 | 10.1 | 0.9 | - | - | 7 |
| Podarcis muralis | no | Asturias (Spain) | high | high | high | 65 | 12 | 3.0 | 0.7 | - | 1.1 | 4 |
| Podarcis muralis | no | Dereköy (Turkey) | high | high | high | 69* | - | 14.0 | 0.9 | - | 0.5^+^ | 14 |
| Podarcis peloponnesiaca | no | Peloponnisos (Greece) | high | high | high | 82 | 9 | - | - | - | 1.2 | 15 |
| Podarcis pityusensis | yes | Ibiza island (Spain) | high | low | low | 82 | 21 | 18.0 | 1.0 | - | 0.7 | 7 |
| Podarcis siculus | yes | Licosa island (Italy) | low | low | high | >80 | - | 2.2^~^ | 0.6 | - | - | 16 |
| Podarcis siculus | no | Captivity | - | - | - | 90 | 12 | 3.7 | 0.8 | - | - | 4, 7 |
| Podarcis siculus | no | Punta Licosa (Italy) | high | high | high | 78 | - | 1.6^~^ | 0.5 | - | - | 16 |
| Podarcis siculus | no | Filyos (Italy) | high | high | high | 85* | - | 12.0 | 0.9 | - | 0.9 | 17 |
| Podarcis tauricus | no | Serbia | high | high | high | 70 | 10 | 7.0 | 0.9 | 0.5 | 1.1 | 18, 19 |
| Podarcis tauricus | yes | Ionian islands (Greece) | high | high | high | 74 | 9 | - | - | - | 0.9 | 20 |
| Podarcis tiliguertus | yes | Captivity | - | - | - | 87 | - | 15.0 | 0.9 | - | - | 7 |

**^+^Only lowland population**

**^~^Mean Life Expectancy**

***Asymptotic size**

**References:**

1. Galán, P. (1999). Demography and population dynamics of the lacertid lizard Podarcis bocagei in north-west Spain. Journal of Zoology, 249(2), 203-218. / Galán, P. (1986). Morfología y distribución del género Podarcis Wagler, 1830 (Sauria, Lacertidae) en el noroeste de la Península Ibérica. Rev. Esp. Herp, 1, 85-142.

2. Galán, P. (2004). Structure of a population of the lizard Podarcis bocagei in northwest Spain: variations in age distribution, size distribution and sex ratio. Animal biology, 54(1), 57-75.

3. Galán, P. (2008). Ontogenetic and sexual variation in the coloration of the lacertid lizards Iberolacerta monticola and Podarcis bocagei. Do the females prefer the greener males?. Animal Biology, 58(2), 173-198.

4. Meiri, S. (2008). Evolution and ecology of lizard body sizes. Global Ecology and Biogeography, 17(6), 724-734.

5. Bauwens, Dirk, and Ramon Diaz-Uriarte. "Covariation of life-history traits in lacertid lizards: a comparative study." The American Naturalist 149.1 (1997): 91-111.

6. Sá‐Sousa, P., Almeida, A. P., Rosa, H., Vicente, L., & Crespo, E. G. (2000). Genetic and morphological relationships of the Berlenga wall lizard (Podarcis bocagei berlengensis: Lacertidae).

7. Sabath, N., Itescu, Y., Feldman, A., Meiri, S., Mayrose, I., & Valenzuela, N. (2016). Sex determination, longevity, and the birth and death of reptilian species. Ecology and Evolution, 6(15),

8. Galán, P. (2003). Reproductive characteristics of an insular population of the lizard Podarcis hispanica from Northwest Spain (Cies Islands, Galicia). Copeia, 2003(3), 657-665.

9. Castilla, A. M., & Bauwens, D. (2000). Reproductive characteristics of the island lacertid lizard Podarcis lilfordi. Journal of Herpetology, 390-396.

10. Rotger, A., Igual, J. M., Genovart, M., Rodríguez, V., Ramon, C., Pérez-Mellado, V., ... & Tavecchia, G. (2021). Contrasting Adult Body-Size in Sister Populations of the Balearic Lizard, Podarcis lilfordi (Günther 1874) Suggests Anthropogenic Selective Pressures. Herpetological Monographs, 35(1), 53-64.

11. Carretero, M. Á., & Salvador Milla, A. (2016). Lagartija parda–Podarcis liolepis (Boulenger, 1905).

12. Adamopoulou, C., & Valakos, E. D. (2000). Small clutch size in a Mediterranean endemic lacertid (Podarcis milensis). Copeia, 2000(2), 610-614.

13. Meiri, S. (2007). Size evolution in island lizards. Global Ecology and Biogeography, 16(6), 702-708.

14. Eroğlu, A. İ., Bülbül, U., Kurnaz, M., & Odabaş, Y. (2018). Age and growth of the common wall lizard, Podarcis muralis (Laurenti, 1768). Animal Biology, 68(2), 147-159.

15. Maragou, P. (1999). Comparative data on reproduction in Podarcis erhardii, Podarcis peloponnesiaca, and Podarcis taurica (Reptilia, Sauria, Lacertidae). Israel Journal of Ecology and Evolution, 45(4), 487-496.

16. Raia, P., Guarino, F. M., Turano, M., Polese, G., Rippa, D., Carotenuto, F., ... & Fulgione, D. (2010). The blue lizard spandrel and the island syndrome. BMC Evolutionary Biology, 10(1), 1-16.

17. Eroğlu, A. İ., Bülbül, U., & Kurnaz, M. (2017). Age structure and growth in a Turkish population of the Italian wall lizard Podarcis siculus (Rafinesque-Schmaltz, 1810)(Reptilia: Lacertidae).

18. Altunýþýk, A., Kalayci, T. E., Uysal, Ý., Tosunoðlu, M., & Özdemir, N. (2016). AGE, ADULT SURVIVAL RATE, AND ADULT LIFE EXPECTANCY OF A Podarcis tauricus POPULATION (REPTILIA: LACERTIDAE) FROM SAROS BAY, TURKEY. Russian Journal of Herpetology, 23(4).

19. Yıldırım E, Kumlutaş Y, Candan K, Ilgaz Ç, 2021. The Study on the Relationships Between the Age Structure and Body Size of the Bridled Skink, Heremites vittatus, (Oliver, 1804) from different Altitudes in Turkey. Journal of the Institute of Science and Technology, 11(2): 906-915.

20. Chondropoulos, B. P., & Lykakis, J. J. (1983). Ecology of the Balkan wall lizard, Podarcis taurica ionica (Sauria: Lacertidae) from Greece. Copeia, 991-1001.
